# Immunological analysis of hybrid neoantigen peptide encompassing class I/II neoepitope-pulsed dendritic cell vaccine

**DOI:** 10.3389/fimmu.2023.1223331

**Published:** 2023-10-10

**Authors:** Shinji Morisaki, Hideya Onishi, Takafumi Morisaki, Makoto Kubo, Masayo Umebayashi, Hiroto Tanaka, Norihiro Koya, Shinichiro Nakagawa, Kenta Tsujimura, Sachiko Yoshimura, Poh Yin Yew, Kazuma Kiyotani, Yusuke Nakamura, Masafumi Nakamura, Takanari Kitazono, Takashi Morisaki

**Affiliations:** ^1^ Fukuoka General Cancer Clinic, Fukuoka, Japan; ^2^ Department of Cancer Therapy and Research, Graduate School of Medical Sciences, Kyushu University, Fukuoka, Japan; ^3^ Department of Medicine and Clinical Science, Graduate School of Medical Sciences, Kyushu University, Fukuoka, Japan; ^4^ Department of Surgery and Oncology, Graduate School of Medical Sciences, Kyushu University, Fukuoka, Japan; ^5^ Corporate Headquarters, Cancer Precision Medicine Inc., Kawasaki, Japan; ^6^ Cancer Precision Medicine Center, Japanese Foundation for Cancer Research, Tokyo, Japan; ^7^ National Institutes of Biomedical Innovation, Health and Nutrition, Ibaraki, Osaka, Japan

**Keywords:** neoantigen, neoepitope, dendritic cell, vaccine, antigen presentation

## Abstract

Neoantigens/ are tumor-specific antigens that evade central immune tolerance mechanisms in the thymus. Long-term tumor-specific cytotoxic T lymphocyte activity maintenance requires class II antigen-reactive CD4^+^ T cells. We had previously shown that intranodal vaccination with class I neoantigen peptide-pulsed dendritic cells (DCs) induced a robust immune response in a subset of patients with metastatic cancer. The present study aimed to perform a detailed *ex vivo* analysis of immune responses in four patients receiving an intranodal hybrid human leukocyte antigen class II neoantigen peptide encompassing a class I neoantigen epitope (hybrid neoantigen)-pulsed DC vaccine. After vaccination, strong T-cell reactions against the hybrid class II peptide and the class I-binding neoantigen peptide were observed in all four patients. We found that hybrid class II neoantigen peptide-pulsed DCs stimulated CD4^+^ T cells via direct antigen presentation and CD8^+^ T cells via cross-presentation. Further, we demonstrated that hybrid class II peptides encompassing multiple class I neoantigen epitope-pulsed DCs could present multiple class I peptides to CD8^+^ T cells via cross-presentation. Our findings provide insight into the mechanisms underlying hybrid neoantigen-pulsed DC vaccine therapy and suggest future neoantigen vaccine design.

## Introduction

Tumor-specific antigens recognized by the T-cell receptors of cytotoxic T lymphocytes (CTLs) are antigen epitopes bound to human leukocyte antigen (HLA) class I on the surface of tumor cells and are usually short peptides 8–11 amino acids in length (1, 2). Among the tumor-specific antigens, neoantigens, which have a newly generated amino acid sequence due to a genetic mutation in tumor cells, are the most tumor-specific and potent, since they circumvent the central immune tolerance (3–5). Neoantigens and the CTLs that respond to them are essential for the efficacy of immune checkpoint inhibitors (6–10). Therefore, clinical trials have been initiated to predict and synthesize neoantigens, by genetic analysis of tumors and analysis of HLA class I affinity, and to use them as materials for cancer vaccines, of which the safety and efficacy have already been reported (11–15). Recently, a combination of neoantigen vaccines and immune checkpoint inhibitors has been reported to help treat various cancers (16).

We initiated neoantigen peptide-pulsed dendritic cell (DC) vaccine therapy involving the generation of a neoantigen profile of each patient’s tumor using the neoantigen prediction pipeline, followed by the selection and synthesis of a short peptide with strong HLA class I affinity and use of neoantigen peptides for pulsing the patients’ monocyte-derived DCs; the efficacy results have already been reported (17–20). DC vaccines have been shown to be the most safe and effective cancer vaccines (21, 22), and our method of intranodal administration of cell vaccines was based on a scientific rationale (23). We reported an association between the efficacy of neoantigen DC vaccine monotherapy and increased immune response of peripheral blood lymphocytes to neoantigen epitopes after vaccine administration, including a complete durable response in a case of renal cell carcinoma with lung metastases (20).

Many previous studies have shown that vaccines targeting class I affinity peptide-specific CTLs alone cannot maintain adequate antitumor immunity (24, 25). CD4^+^ T cells are known to be essential for long-term CTL activation (26). In this process, CD4^+^ T cells are not only important for cytokine production as described in the old model, but also for sustained stimulation of CTLs via DC activation (27). In particular, the activation of CTLs and CD4^+^ T cells by the same antigen-presenting cells (APCs) has been reported to be significant (28). Therefore, a neoantigen vaccine that simultaneously activates CD4^+^ T cells and class I-restricted antigen-specific CTLs is ideal.

We proposed that a class II-affinity neoantigen peptide containing a class I-restricted neoantigen epitope can activate class I-restricted killer T cells and class II-restricted helper T cells. In this study, we referred to it as a hybrid neoantigen peptide. When taken up by DCs, this hybrid neoantigen peptide likely activates CD4^+^ T cells that respond to class II-affinity neoantigens and CD8^+^ killer T cells that respond to class I-restricted neoantigens through the cross-presentation function of DCs.

We initiated a vaccine regimen using a neoantigen peptide-pulsed DC vaccine, in combination with class I and class II-affinity neoantigen peptides, in cases where class II (HLA-DR)-restricted neoantigen peptides could be predicted. We encountered cases where the long peptide also contained a neoepitope with a strong affinity for class I, resulting in high efficacy and immune response. In such cases, the long peptides were revealed to be hybrid neoantigens that could activate CD4^+^ T cells and CD8^+^ CTLs by cross-presenting the encapsulated class I epitope via DCs.

In this *ex vivo* study, we aimed to validate the immunological analysis of hybrid neoantigen peptide-pulsed DC vaccines using immune cells from patients treated with the vaccine. We believe that our report, demonstrating the immunological effects of neoantigen vaccines, will help in the future design and clinical potential of the vaccines.

## Materials and methods

### Patients and neo-p-DC vaccine therapy

**Figure 1:**
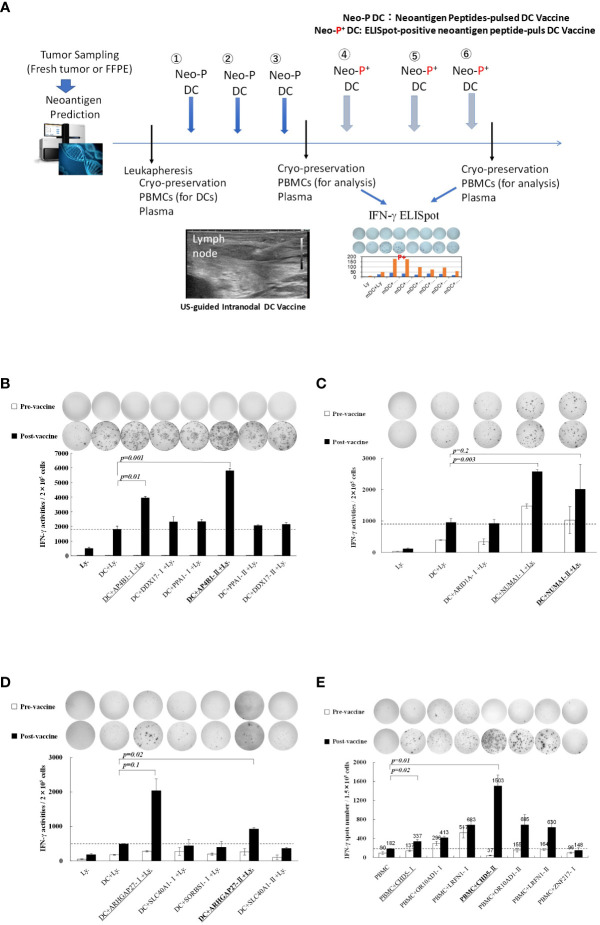
**(A)** Protocol for intranodal neoantigen peptide-pulsed dendritic cell (DC) vaccine therapyPrior to treatment, tumor sampling (fresh tumor tissue or formalin-fixed paraffin-embedded tissue), genetic testing for neoantigen prediction via next-generation sequencing, leukapheresis, and synthesis of neoantigen peptides (selection and synthesis of HLA class I **(A, B, C)** and class II HLA-DRB1 affinity peptides) were performed. Monocyte-derived DCs were cultured and administered to patients (intranodal DC vaccination) after confirming their sterility and quality (FACS analysis). After the administration of three vaccines at two-week intervals, IFN-γ ELISpot analysis was performed, and an ELISpot-positive neoantigen peptide was used for the following three DC vaccines. After administering the six DC vaccines, ELISpot responses were measured again. Peripheral PBMCs and plasma were cryopreserved before, during, and after treatment. **(B–D)** Immune responses of peripheral blood lymphocytes to neoantigen peptides before and after intranodal neoantigen DC vaccine **(B–E)** Each panel shows the IFN-γ ELISpot response to neoantigen peptide measured in peripheral blood lymphocytes from each patient before and after three vaccine cycles [Ly., lymphocytes alone; DC + Ly, dendritic cells + lymphocytes; DC + peptide + Ly., dendritic cells + neoantigen peptide-I (class-I peptide) or neoantigen peptide-II (class II peptide) + lymphocytes); Bold and underlined peptide: hybrid class-II peptide encompassing class I neoepitope; underlined peptide: class-I peptide encompassed within that hybrid peptide **(B–E)**, Data from Patients 1, 2, 3, and 4, respectively. Each panel of the ELISpot analysis used the intensity and size of every spot counted in the well after calculating the spot number for every well. For each spot, the intensity and size were multiplied, and the values of all spots were summed. Finally, the results were divided by 1,000 to obtain the activity value in **(B–D)**. All measurements were performed in triplicate. Data are represented as mean ± SD. Each ELISpot figure is representative of a triplicate assay.

**Table 1** shows the clinical profiles of patients undergoing the intranodal hybrid neoantigen peptide-pulsed DC vaccine therapy who consented to immunological analysis. The study included one patient each with the following cancers: ovarian, esophageal, colorectal, and fibrosarcoma. Four patients had stage IV disease. **Figure 1A** illustrates the protocol for intranodal DC vaccine therapy. Leukapheresis was performed before therapy, and peripheral blood mononuclear cells (PBMCs) were cryopreserved. The DC culture was performed as described below. The first half of the vaccine was administered thrice at 7- to 14-day intervals (intranodal injection guided by ultrasonography). After the third vaccination, peripheral PBMCs were isolated to determine the immune response to each neoantigen peptide using ELISpot reactions. The second half of the vaccine was administered three times at 3-week intervals, and tumor responses were imaged using computed tomography (CT) after completion. PBMCs and plasma were separated and collected before treatment, during vaccine therapy, and after completion of the six vaccines and frozen for subsequent peripheral blood lymphocyte immune response analysis.

**Table 1:** Clinical profiles of the study participants.

Patient No.	Gender/Age	Tumor	Metastasis	Stage	Sample (fresh or FFPE)	Prior therapy	Combined therapy	Response	Duration (months)
1	M/73	Esophagus	Liver and LNs	IV	Fresh origin tumor	Chemotherapy	None	PR	6
2	F/48	Ovarian	Peritoneum	IV	Fresh (peritoneum)	Surgery and chemotherapy	None	SD	18
3	M/69	Colon	Liver and peritoneum	IV	Origin a tumor (FFPE)	Surgery and chemotherapy	Chemotherapy	PR	26
4	F/52	Sarcoma	Lung, liver, and bone	IV	Liver metastasis (FFPE)	Surgery, chemotherapy, and radiation	Chemotherapy	SD	6

### Neoantigen prediction

HLA class I genotypes in patients were predicted from normal whole-exome sequencing data using OptiType (29), whereas HLA class II genotypes were predicted using PHLAT (30). Neoantigens were predicted for each non-synonymous variant, and the binding affinities of all possible 8–11-mer peptides for HLA-A and HLA-B were examined using NetMHC v3.4 and NetMHCpanv2.8, as previously described (17, 18). In contrast, the binding affinities of all possible 15–18-mer peptides for HLA-DRB1 were examined using netMHCII-2.2 and netMHCIIpan-3.1 (18, 31).

Candidate neoantigen peptides with a predicted binding affinity (IC_50_) of no more than 50 nM were selected for further analysis(An exception was made for research peptides with binding affinities of 2-331 nM). The Cancer Genome Atlas (TCGA) mRNA expression data for colon carcinoma and TCGA mRNA data for breast carcinoma were considered for selecting potential neoantigen candidates.

For each patient, two to six class I peptides and one to three class II peptides were selected from those with a high affinity for HLA class I (A, B, and C) and HLA-DRB1, respectively. Peptides were synthesized, and their quality was confirmed by HPLC analysis. **Table 2** shows the amino acid sequence, affinity, and mRNA expression of the class II hybrid neoantigen peptide used for the four patients in this study. Patient 2 had only one class I neoantigen epitope, whereas patients 1, 3, and 4 had multiple class I neoantigen epitopes. Class I neoantigen peptides with the highest affinity were selected for therapeutic use.

**Table 2:** Amino acid sequence of hybrid class II neoantigens and their encompassing class I neoepitopes.

Patient No	Nonsynonymosmutation No.	Neoantigen No.	Hybrid Class-II neoantigen underlined; encompassing Class-I peptide, bald; amino acid substituion	HLA-DR, affinity, mRNA	Class-I neoepitope	Class-I, affinity
1	76	281	**AP4BI** QKKLVYLYMCTCAPLKPD	HLA-DRB1:0901 affinity;24 mRNA;20	**AP4BI** YLYMCTCAPL YMCTCAPL TCAPLKPD LYMCTCAPL	HLA02:01, affinity; 5 HLA-B15:01, affinity 81 HLA-C03:33, affinity 3 HLA-C03:04, affinity 303
2	32	134	**NUMA1** REVAWLTQERGRAQLA	HLA-DRB1:1101 affinity; 331 mRNA 29	**NUMA1** EVAWLTQERGR	HLA-A2603, afinity;21
3	149	364	**ARHGAP27** PLFPFLHFRQFIAAIKLQ	HLA-DRB1:1201 affinity;4 mRNA;1960 (TCGA median))	**ARHGAP27** FPFLHFRQFIA PLFPFLHFR FPFLHFRQ FPFLHFRQFI FPFLHFRQF	HLA-B54:01, affinity 2 HLA-A11:01. affinity 178 HLA-B54:01, affinity 27 HLA-B54:01, affinity 32 HLA-B54:01, affinity 43
4	17	33	**CHD5** EFSFEYNAIRSGKKVFRM	HLA-DRB1:1201 affinity;15, mRNA:80	**CHD5** EFSFEYNAIR FSFEYNAIR EYNAIRSGK SFEYNAIR FSFEYNAI	HLA-A33:03, affinity:44 HLA-A33:03, affinity62 HLA-A33:03, affinity 146 HLA-A33:03, affinity 323 HLA-C03:04, affinitty 31

### DC vaccine culture and administration

DC vaccines were cultured as previously described (19, 20). Briefly, PBMCs obtained by leukapheresis prior to treatment were cryopreserved, thawed, and seeded (2 × 10^6^ mononuclear cells per well in 2 mL of medium) in complete medium containing 1% autologous serum in 6-well plates (FALCON, Franklin Lake, NJ, USA) for 30 min. After removing floating cells and washing with RPMI, adherent cells were cultured in DC complete medium containing 100 ng/mL GM-CSF (Primmune Inc., Kobe, Japan) and 50 ng/mL IL-4 (Primmune). On day 6 of culture, maturation factors (MFs) containing 500 IU/mL TNF-α (PeproTech Inc., Rocky Hill, NJ, USA) and 500 IU/mL IFN-α (Dainippon Pharma) were added. Neoantigen peptides were dissolved in sterile water containing DMSO before use, filtered through a 0.22 μm Millipore syringe (Millipore, Mosheim, France), and finally tested for endotoxin, β-glucan, and mycoplasma, which were below detection limits. Endotoxin and β-glucan were analyzed using a Toxinometer ET-600 (Wako Pure Chemical Industries, Ltd., Osaka, Japan). Mycoplasma contamination was detected using a MycoAlert (Lonza Rockland Inc. Rockland ME, USA). Class I short peptides were added to DCs after adding MFs, and class II long peptides were added before the addition of MFs. The supernatants of DC cultures were collected and stored (4°C) for subsequent exosome isolation, followed by the extraction of dexosomes, as described below. Endotoxin, β-glucan, and mycoplasma contamination of the culture medium was tested before the collection of mDCs and were found to be below the detection limits. FACS analysis of DCs revealed that they were positive for HLA-DR, HLA class I, CD86, and CD40 and negative for CD14. Cell counts were performed using the Sysmex CD-500 system (Sysmex Corp., Kobe, Japan). DCs were suspended in saline (0.5 mL) and administered to patients by a skilled physician in a 1 mL syringe with a 25 G needle under ultrasound guidance, targeting the cortical-medullary border of normal inguinal lymph nodes. The procedure details have been previously described (19, 20, 23).

### ELISpot response

ELISpot analysis was performed using the Human IFN-γ ELISpot^plus^ kit (MABTECH, Cincinnati, OH, USA), according to the manufacturer’s instructions. Briefly, 96-well plates were washed four times with sterile PBS (200 μL) and preconditioned with medium (200 μL) containing 10% of the autologous serum used for cell suspension for 30 min–1 h. The medium was removed, and immune cells, peptides, or exosomes were added using the following assay methods. After incubation for a certain period, the plate was washed five times with PBS (200 μL), the detection antibody (7-B6-1-biotin) was dissolved (1 μg/mL) in 0.5% fetal bovine serum-containing PBS (PBS-0.5% FCS), and the solution (100 μL/well) was added and incubated for 2 h at approximately 15–25 °C. The plate was washed five times with PBS (200 µL), the secondary antibody (Streptavidin-HRP) was dissolved in PBS-0.5% FCS at a ratio of 1:1000, and the antibody solution (100 µL/well) was added and incubated at approximately 15–25°C for 1 h. The plate was then washed five times with PBS (200 μL) to remove the internal solution, and 100 μL/well of TMB substrate solution was added and allowed to stand for 10 min. The plates were finally washed five times with deionized water to stop the reaction, wells were dried, and the spots were detected and analyzed using an automated ELISpot reader 0.8 classics (AID GmbH, Strasberg, Germany).

We performed immunological analysis of lymphocytes before and after neoantigen peptide-pulsed DC vaccine using the ELISpot assay. Briefly, frozen PBMCs, which had been cryopreserved before therapy from each patient, were thawed and seeded in a 6-well plate at a density of 2 × 10^6^ cells/mL and cultured at 37°C for 30 min. The monocytes were allowed to adhere, non-adherent lymphocytes were removed, and a DC medium containing GM-CSF + IL-4 was added, followed by culture for 5 days. When the cells became immature, DC-like, and floating, they were collected and spread onto ELISpot plates at a density of 5 × 10^3^ cells/well. The HLA class I peptide was added after the maturation of DCs, and the HLA class-II peptide was added before the maturation of DCs. Lymphocytes isolated from the cryopreserved PBMCs obtained before and after three vaccine doses were added to DC cultures at 1.5 × 10^5^ cells/well and cultured for 48 h for ELISpot analysis. PBMCs were added at 2 × 10^5^ cells/well density. Peptides were added similarly, and the ELISpot reaction was analyzed after 48 h of incubation.

For some ELISpot analyses, the ELISpot activity value of all wells was used to determine the intensity and size of every counted spot in the well. The intensity and size of each spot were multiplied to determine ELISpot activity. The values of all spots were summed, and the results were divided by 1,000 to obtain the activity value.

### Isolation of CD8^+^ T cells and CD4^+^ T cells

CD8^+^ or CD4^+^ T cells were obtained by positive selection of patient PBMCs using anti-human CD8 or anti-CD4 antibody-coated microbeads, following the manufacturer’s instructions (MACS Beads Cell Separation Kit [Miltenyi Biotech, Bergisch Gladbach, Germany]). The purity of CD8^+^ cells was assessed using flow cytometry and was found to be > 98%; however, the purity of CD4^+^ cells was found to be relatively low (approximately 90%).

### Culture of hybrid peptide-pulsed DC-stimulated lymphocytes and their reactivity to class-I or class-II neoantigen peptides

We examined whether the hybrid peptides encompassing class I neoantigen epitope-pulsed DCs could restimulate CD8^+^ and/or CD4^+^ T cells. Autologous lymphocytes obtained after three cycles of neoantigen DC vaccines were restimulated with mature (m)DCs pulsed with hybrid neoantigen peptides or control non-immunogenic peptides and cultured for 7 days. Lymphocytes were collected, and a positive selection of CD8^+^ or CD4^+^ T cells was performed using CD8-positive or CD4-positive separation beads (MACS beads). Autologous immature (im)DCs (5 × 10^3^ cells/well) were seeded in 96-well ELISpot plates, and the hybrid peptide encompassing class I peptide was added to the DCs in 96-well ELISpot plates after maturation. For further analysis of the hybrid class II long peptides, the latter were added to imDCs before maturation. The separated CD8^+^ T lymphocytes or CD4^+^ T cells (each 1.5 × 10^5^ cells/well) were added to the mDCs and cultured for 48 h for the ELISpot reaction.

### Ethics

Cell processing, neoantigen examination, immunotherapy procedures, and immunological analysis were approved by the ethics committee of our institution (FGCC-EC009), with the patients’ written informed consent for the procedure based on type 3 regenerative medicine under the Japanese Law for Ensuring the Safety of Regenerative Medicine.

### Statistical analysis

Statistical analyses were performed using GraphPad Prism, version 8 (GraphPad Software Inc., San Diego, CA, USA). Data are presented as mean ± standard deviation (SD). Student’s *t*-test was used to compare continuous variables between the two groups. Statistical significance was set at *p* < 0.05.

## Results

### Immune responses after intranodal neoantigen peptide-pulsed DC vaccine administration

Detailed immunological analysis was performed on four patients treated with the hybrid neoantigen DC vaccine. **Figures 1B–E** shows the results of IFN--ELISpot assays for each neoantigen peptide using peripheral blood lymphocytes isolated from the four patients before and after three vaccine doses. Although the degree of enhancement of lymphocyte response to each neoantigen peptide differed across patients, the number of T cells reacting to neoantigens increased compared to that before vaccination in all four cases. For example, in patient 1 (**Figure 1B**, upper left), almost no immunoreactivity to peptides was observed before vaccination (white column). However, strong reactions were observed against the AP4B1-mutant(m) peptide with high affinity to the HLA class II molecule (AP4B1-IIm) about 3-fold increase over baseline and against the AP4B1(m) short peptide with high affinity to HLA class I molecules (AP4B1-Im) about 2-fold increase over baseline after vaccination (black column). AP4B1-II (m) is a long peptide with 17 amino acids incorporating AP4B1-I (m) (**Table 2**). In patients 1 and 4, the number of T lymphocytes reacting to the neoantigen peptide, in response to the class II-restricted mutant peptide, was higher than that in response to the class I-restricted mutant peptide (**Figures 1B, E**). In patients 2 and 3, the response to the class I-binding peptide, whose amino acid sequence was included within the class IIm peptide, was higher than that to the class II peptide (**Figures 1C, D**). Our results suggests that neoantigen vaccines increase the number of T cells that respond to both class I and II neoepitopes. And that the response to Class I or II neoepitopes is patient-specific, differing between patients depending on their tumor type.

Further, we discovered that post-vaccine peripheral blood lymphocytes in all cases increased the number of IFN-producing cells in both the presence and absence of *in vitro* stimulation of DCs pulsed with the neoantigen peptide, indicating the presence of activated T cells in circulating peripheral blood (data from lymphocytes alone; left side in each panel).

### Hybrid neoantigen peptide-pulsed DCs cross-presented class-I neoantigen epitopes to CD8 T cells

The class II neoantigen peptides used for the four patients were long peptides expected to have high affinity for a class II molecule (HLA-DRB1) and were predicted to include a class I-binding neoantigen epitope. **Table 2** shows the amino acid sequence of the class II hybrid peptides encompassing the class I neoantigen epitope, which was likely to have a binding affinity toward HLA class I molecules. Although patients 1, 3, and 4 harbored a class II hybrid peptide encompassing multiple class I-binding peptides, the hybrid neoantigen peptides from Patient 2 harbored only one class I neoantigen epitope.

**Figure 2:**
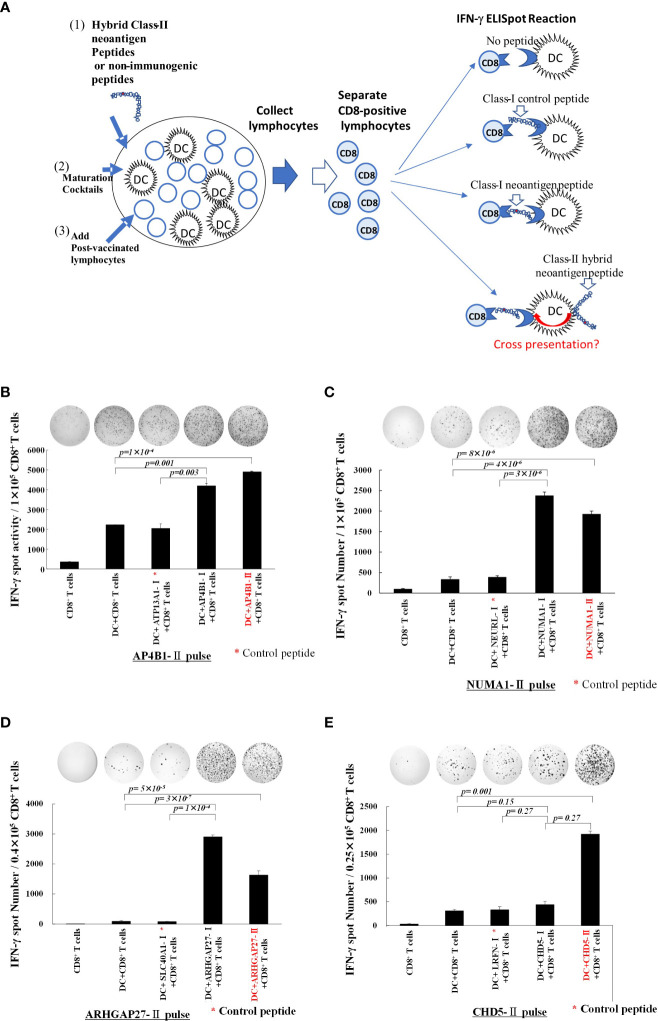
Class-II hybrid neoantigen encompassing a class I neoantigen epitope-pulsed dendritic cells (DCs) cross-present neoantigen to CD8^+^ T cells **(A)** An experimental schema for the investigation of the cross-presentation of class I epitopes by DCs pulsed with class II neoantigen peptides. **(B–E)** Data from Patients 1, 2, 3, and 4, respectively. Mature DCs pulsed with class II hybrid peptides encompassing a class I neoepitope and post-vaccination peripheral blood lymphocytes from each patient were co-cultured for 7 days. CD8^+^ T cells were positively selected and restimulated with mDCs pulsed with class I or II hybrid peptides. Controls included CD8^+^ T cells alone, CD8^+^ T cells + non-peptide-pulsed mDCs, and CD8^+^ T cells + non-immunogenic control peptide-pulsed mDC. * Non-immunogenic control peptides are shown in each of **(B–E)** panels. Positively selected CD8^+^ T cells cultured with class II neoantigen peptide plus DCs were allowed to react with class I neoantigen-epitope-pulsed DCs or class II peptide-pulsed DCs for comparison. Results of the ELISpot reaction over 48 h are shown. CD8^+^ T cells exhibited immunoreactivity toward class I neoantigen peptide-pulsed mDCs and class II neoantigen peptide-pulsed mDCs. All measurements were performed in triplicate. Data are represented as mean ± SD. Each ELISpot figure is representative of a triplicate assay.

To examine whether hybrid class II long peptide-pulsed DCs can cross-present class-I neoepitopes, we designed the experiment as shown in **Figure 2A**. Peripheral blood lymphocytes obtained from each patient after three rounds of vaccination were co-cultured with monocyte-derived DCs pulsed with hybrid neoantigen peptides following maturation for 7 days, and then CD8^+^ T cells were positively selected. CD8^+^ T cells were restimulated with mDCs pulsed with or without a non-immunogenic control peptide or neoantigen epitope peptide. As shown in **Figures 2B–E**, CD8^+^ T cells co-cultured with mDCs pulsed with the class I neoepitope peptide contained in the hybrid peptide exhibited significantly higher immunoreactivity than T cells cultured alone, without peptide-pulsed DCs, or with control class I peptide-pulsed DCs. To further confirm that CD8^+^ T lymphocytes sensitized with hybrid neoantigens can recognize the cross-presented neoantigen epitope, we cultured CD8^+^ T cells with DCs pulsed with the hybrid neoantigen long peptide or its class I neoepitope. In the four patients, CD8^+^ T cells reacted with hybrid neoantigen long peptides, indicating cross-presentation by the DCs.

### Clinical efficacy and adverse reactions

Three patients (1, 3, and 4) received concomitant chemotherapeutic agents. Therefore, it was impossible to evaluate the clinical efficacy of neoantigen DC vaccines alone. Patient 2, who received only DC vaccine therapy, remained in SD without the progression of peritoneal dissemination. Patient 4 with fibrous sarcoma remained stable, except for liver metastases, which were consequently stabilized with multimodality therapy (radiation, chemotherapy, and immune therapy).

**Figure 3:**
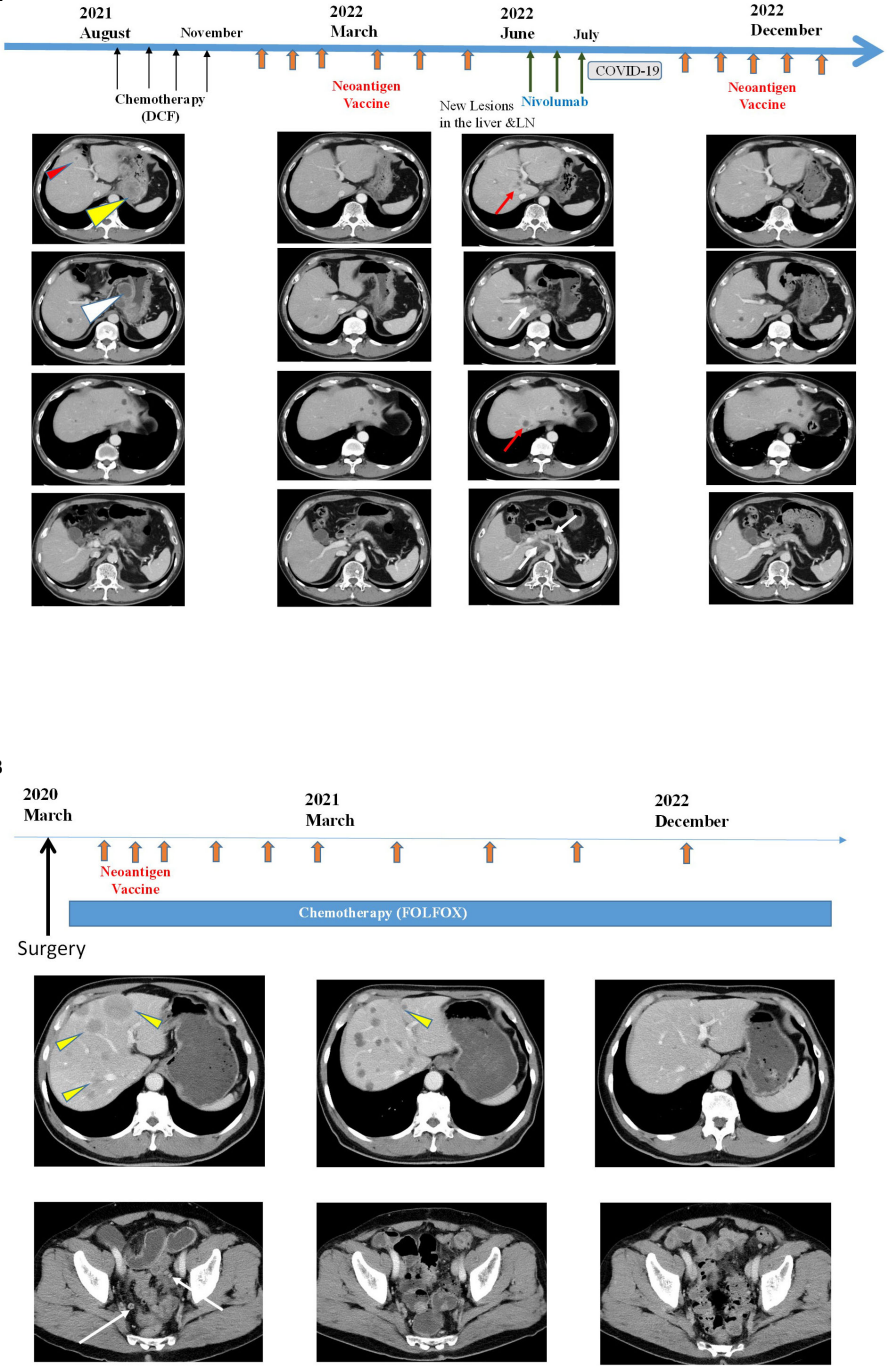
Clinical responses after intranodal hybrid neoantigen DC vaccine **(A)** Clinical course and computed tomography (CT) of Patient 1. Data before vaccination (August 2021) and after six vaccination cycles (March 2022, June 2022, and December 2022). Thin red triangles indicate liver metastases, yellow triangles indicate original large tumors, and white triangles indicate multiple lymph node metastases. The red arrow indicates new liver metastasis, and the white arrows indicate new LN metastasis. In the right panels, both liver and LN metastases disappeared. **(B)** Clinical course and CT image of Patient 2. CT scans were performed before vaccination (March 2020), at March 2021 and 33 months after vaccination initiation (December 2022). Yellow triangles indicate multiple liver metastases, and the white arrows indicate peritoneal metastases. Almost all tumors disappeared by December 2022.

Imaging data for the two patients (Patients 1 and 3) who had sustained tumor shrinkage are shown in **Figure 3**. Patient 1 had squamous cell carcinoma of the esophagogastric junction with multiple lymph nodes and liver metastases. Before treatment initiation, the patient had a large primary lesion, multiple lymph node metastases, and small liver metastases; however, after 10 months of treatment, most of the lesions had shrunk. However, new liver and lymph node metastases appeared in June 2022; therefore, three doses of nivolumab therapy were initiated in June 2022 but were eventually discontinued due to mild immune-related adverse events. Finally, the liver metastases and lymph nodes almost disappeared, as per CT in December 2022 (**Figure 3A**).

Patient 3 had the most extended treatment history. Patient 3 was a 69-year-old man diagnosed with right-sided colorectal cancer with multiple liver metastases and peritoneal dissemination in February 2020. He started chemotherapy (capecitabine + oxaliplatin + bevacizumab) after right-sided colon resection. The intranodal hybrid neoantigen peptide-pulsed DC vaccine therapy was initiated in October 2020. The patient is still undergoing treatment, has multiple liver metastases, and has a durable progression-free response (**Figure 3B**). The immunological analysis of all four patients showed no vaccine-related immunological adverse events. All patients remained under appropriate care and treatment for 12–28 months after the beginning of neoantigen vaccine treatment.

### Class II neoantigen peptide encompassing the class-I epitope-pulsed DCs activated CD8^+^ and CD4^+^ T cells

**Figure 4:**
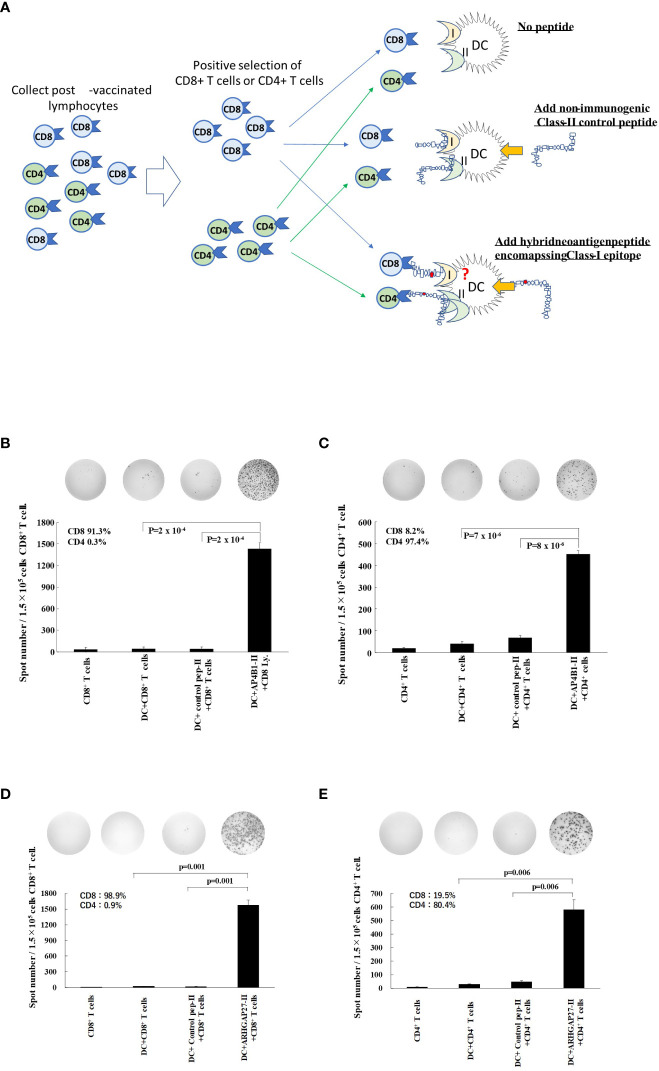
Hybrid neoantigen peptide-pulsed DCs restimulate both CD8^+^ and CD4^+^ antigen-reactive T cells **(A)** Background of the experiment. **(B–E)** CD8^+^ T lymphocytes and CD4^+^ T cells were positively selected from post-vaccination PBMCs of Patient 1 and Patient 3, respectively, and co-cultured with DCs pulsed with hybrid class II neoantigen peptide (controls were co-cultured with CD8^+^ or CD4^+^ T cells alone, with DCs without a peptide in each panel, or with DCs pulsed with a non-immunogenic control class II peptide). **(B)** Data of CD8^+^ T cells from Patient 1. **(C)** Data of CD4^+^ T cells from Patient 1. **(D)** Data of CD8^+^ T cells from Patient 3. **(E)** Data of CD4^+^ T cells from Patient 3. CD8^+^ T cells and CD4^+^ T cells are reactive to the hybrid class II peptide-pulsed mDCs. The purity of CD4^+^ T cells of Patient 3 was low (90%) owing to the large number of CD4^+^/CD8^+^ double-positive T cells among CD4^+^ T cells. All measurements were performed in triplicate. Data are represented as mean ± SD. Each ELISpot figure is representative of a triplicate assay.

To further investigate the mechanisms by which the hybrid neoantigen DC vaccine caused immunological and clinical effects in patients 1 and 3, we studied the extent of responses of CD8^+^ T cells and CD4^+^ T cells restimulated by DCs pulsed with class II peptide encompassing the class I epitope in these two patients. The experimental design is shown in **Figure 4A**. As shown in **Figures 4B, C** (4B, CD8^+^ T cells from Patient 1; 4C, CD4^+^ T cells from Patient 1), both CD8^+^ T cells (CD8 purity = 91.3%) and CD4^+^ T cells (CD4 purity = 97.4%) responded to DCs pulsed with class II-binding mutant peptide (AP4BI-IImutant). No ELISpot positivity was observed in CD8^+^/CD4^+^ T cells without DCs, CD8^+^/CD4^+^ T cells with DCs alone, or CD8^+^/CD4^+^ T cells with control peptide-pulsed mDCs. Similar results were obtained in the experiment using post-vaccinated lymphocytes from Patient 3, as shown in **Figures 4D, E**; both CD8^+^ T cells (**Figure 4D**) and CD4^+^ T cells (**Figure 4E**) of Patient 3 responded strongly to the hybrid peptide ARHGAP27-II mutant-pulsed mDCs.

### Class-II neoantigen peptide encompassing the class-I epitope-pulsed DC presented multiple class-I-binding peptides to CD8^+^ T cells

**Figure 5:**
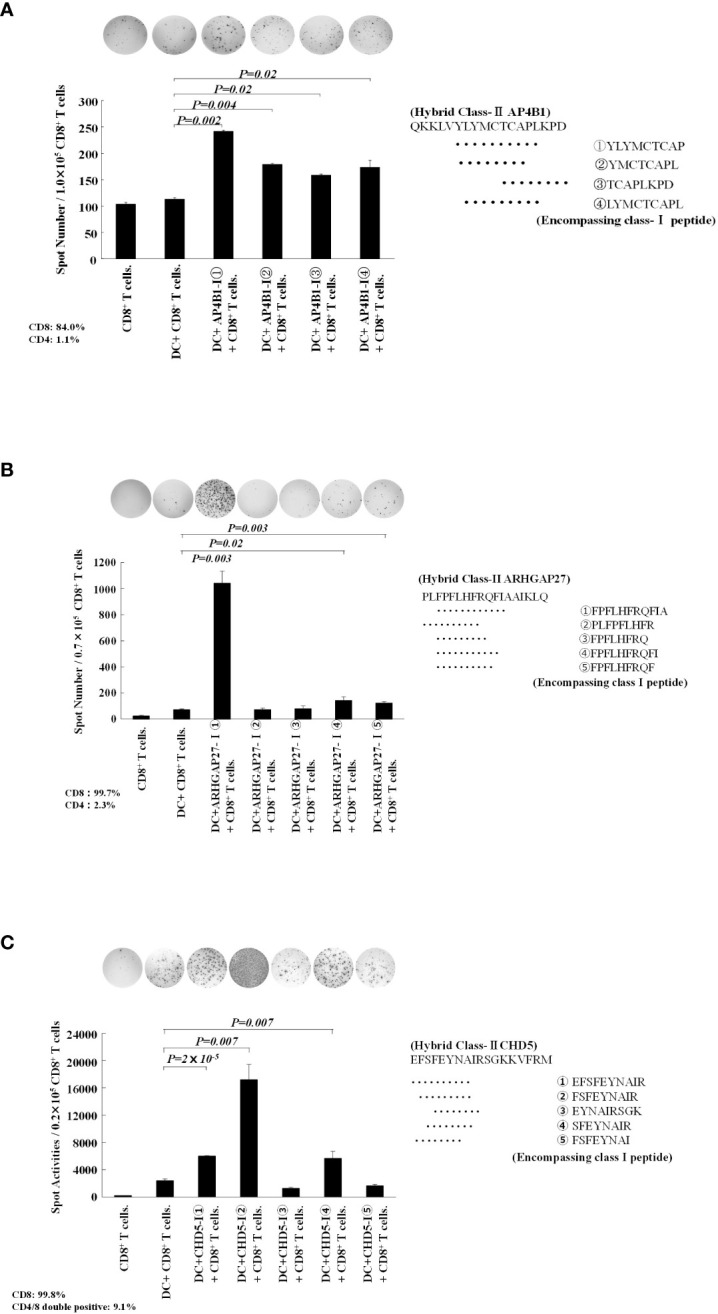
Hybrid neoantigen peptide-pulsed DCs present multiple class-I peptides to CD8 T cells. **(A)** Data of CD8^+^ T cells from Patient 1. CD8^+^ T cells co-cultured with AP4B1-II hybrid peptide-pulsed DCs were reacted with four different class-I-restricted neoantigen peptide-pulsed mDCs, and ELISpot responses were measured. Four peptides induced a CD8^+^ T-cell reaction. **(B)** Data of CD8^+^ T cells from Patient 3. CD8^+^ T cells co-cultured with ARHGAP27-II hybrid peptide-pulsed DCs were reacted with five different class-I-restricted neoantigen peptide-pulsed mDCs, and ELISpot responses were measured. Three peptides induced a CD8^+^ T-cell reaction. **(C)** Data of CD8^+^ T cells from Patient 4. CD8^+^ T cells co-cultured with CHD5-II hybrid peptide-pulsed DCs were reacted with five different class-I-restricted neoantigen peptide-pulsed mDCs, and ELISpot responses were measured. Three peptides induced a CD8^+^ T-cell reaction. All measurements were performed in triplicate. Data are represented as mean ± SD. Each ELISpot figure is representative of a triplicate assay.

In patients 1, 3, and 4, 4–5 kinds of class I neoantigen epitopes were predicted to be included in the hybrid neoantigen peptide (**Table 2**). Hence, we first isolated CD8^+^ T cells from the lymphocytes of Patient 1, which were stimulated and cultured with hybrid neoantigen (AP4B1-IIm)-pulsed DCs, and restimulated them with each of the four predicted class I peptide-pulsed mDCs. ELISPOT analysis showed a high frequency response (2.5 X background) to one peptide, which had predicted high affinity binding to Class I molecules, and lower frequency responses to three other peptides (2, 1.5 and 1.75 X background) (**Figure 5A**). Next, we isolated CD8^+^ T cells from the lymphocytes of Patient 3, which were stimulated and cultured with hybrid neoantigen (ARHGAP27-IIm)-pulsed DCs, and restimulated the cells with each of the five predicted class I peptide-pulsed mDCs. ELISpot analysis showed a high frequency response (20 X background) to one peptide, which had predicted high affinity binding to Class I molecules, and lower frequency responses to two other peptides (3 and 2 X background) (**Figure 5B**). Similar results were obtained when we used post-vaccinated lymphocytes isolated from Patient 4, showing a high frequency response (6 X background) to one peptide, which had predicted high affinity binding to Class I molecules, and lower frequency responses to two other peptides (2 and 2 X background) (**Figure 5C**).

## Discussion

In this study, we performed a comprehensive *ex vivo* analysis of immune cells isolated from patients treated with an intranodal administration of a neoantigen peptide-pulsed DC vaccine, including DCs pulsed with a class II neoantigen peptide encompassing the class I neoantigen epitope as hybrid neoantigens. In the four patients in this study subjected to immunological analysis, we observed a substantial enhancement of the immune response by lymphocytes to the class I and class II peptides after vaccination (**Figure 2**). Our data indicated that the class I/II long peptide activates CD4^+^ T cells as well as CD8^+^ killer T cells suggesting that a portion of the long peptide in DCs is cross-presented on HLA class I molecules, thereby activating CD8^+^ T cells (**Figures 2**, **4**, **5**). Thus, *ex vivo* analysis of post-vaccination immune cells from four patients revealed that DCs, in which a class II hybrid peptide was incorporated, could stimulate CD4^+^ T cells and concomitantly cross-present a class I neoantigen epitope, stimulating antigen-reactive CD8^+^ T cells.

The efficacy of class I-restricted cancer vaccines has also been shown to be limited (24). Unless helper T cells can activate antigen-specific CTLs *in vivo*, complete tumor destruction is unlikely (32, 33). In contrast, complete regression of tumors following the transfer of tumor antigens with class II affinity-specific helper T lymphocytes has been reported. For example, Hunder et al. reported complete tumor regression following the transfer of class II affinity tumor antigen-specific CD4^+^ T cells, although they did not show the precise mechanisms. They speculated that the antitumor effect of antigen-specific CD4^+^ T cells causes antigen spread, resulting in a pronounced antitumor effect (34). Tran et al. reported a case of complete tumor regression in a patient with metastatic cholangiocarcinoma after transferring CD4^+^ T cells grown with a class II affinity peptide specific for ERBB2 mutations (35). Although it was unclear from the results whether the anti-tumor effect of CD4+ T cells was direct or indirect, and whether it was specific to tumors expressing class II antigens, the reports demonstrated the importance of tumor antigen-specific CD4^+^ T cells, which usually have only minor direct cytotoxic activity against tumor cells.

The importance of our findings regarding class I and class II hybrid neoantigen peptide-pulsed DC vaccines is supported by recent studies. Although the neoantigen peptides of HLA class I molecules on tumor cells are recognized by specific T-cell receptors on CD8^+^ killer CTLs, the function of killer T cells alone is insufficient. For example, in the conventional systemic administration of peptides with high affinity for HLA class I molecules, the peptide binds not only to APCs but also to all cells expressing MHC class I molecules, and almost all class I peptides are used in veins (25, 36). Therefore, systemic administration of HLA class I peptides alone is generally ineffective in clinical trials (24). Moreover, even when HLA class I-restricted short peptides bind to APCs, they remain unstable (25). In contrast, once taken up by APCs, long class I peptides are presented to CD8^+^ T lymphocytes via cross-presentation, and the antigen presentation time is suggested to be long and stable (36–40).

The significance of a neoantigen vaccine that can induce both antigen-reactive CD4^+^ and CD8^+^ T cells is supported by many reports, indicating the importance of helper T cells. If the long peptide is also an HLA class II antigen, it has a helper function that enhances the effects of CTLs. Recent studies on cancer peptide vaccines have demonstrated the importance of neoantigens with class II affinity (41, 42). Although cytokine production, such as IL-2 and IFN- γ, is a classical model for the function of CD4^+^ T cells in tumor immunity, the CD40L–CD40 axis that activates DCs through CD4^+^ T cells and the CD70–CD27 axis that activates CTLs through activated DCs constitute a new concept for helper function (26, 43). CD4^+^ T cells have been reported to help downregulate the coinhibitory receptors of CD8^+^ CTLs and promote CTL infiltration into tumors, primarily via the CD70–CD27 axis (43).

We further found that hybrid class II peptides encompassing multiple class I neoantigen epitope-pulsed DCs could present multiple class I peptides to CD8^+^ T cells via cross-presentation, and the processing of longer peptides to shorter class I peptides in DCs may be necessary for inducing higher immune activation (**Figure 5**). When a class II neoantigen peptide encompassing a class I neoantigen epitope (hybrid peptide) is employed in DC vaccines, it not only binds to class II molecules on the DC surface but is also at least partially incorporated into DCs, allowing for regular class I peptide presentation via cross-presentation (40). This suggested that the hybrid neoantigen activates and proliferates antitumor CD4^+^ and CD8^+^ T cells. Thus, neoantigen vaccines would be ideal if they could reliably induce CTLs and helper T cells. Intranodal administration of hybrid neoantigen peptide-pulsed DCs activated CD8^+^ and CD4^+^ T cells responsive to neoantigens with high affinity for class I and II molecules, as illustrated in **Supplementary Figure 1**.

A limitation of our results from this *ex vivo* immune response study is that the relationship between the mechanism of the hybrid neoantigen peptide-pulsed DC vaccine and its efficacy remains unclear. In Patients 1 and 3, in which both tumor neoantigen-specific CD4^+^ and CD8^+^ T cells were activated and proliferated after the hybrid neoantigen-pulsed DC vaccine, a remarkable antitumor effect was sustained. Since the current study did not address the clinical efficacy of the hybrid neoantigen peptide-pulsed DC vaccine alone, further research on the mechanistic determinants of treatment efficacy and clinical trials is warranted.

There are only a few reports on the simultaneous use of class I- and II-binding antigen peptides in vaccines. One study reported that HER-2 peptide vaccine therapy using a synthesized class II peptide encapsulating a class I-binding peptide induced an immune response in CD4^+^ and CD8^+^ T cells (44). In another study, a class II antigen peptide encapsulating the class I antigen epitope of MAGE-A4 was used as a helper/killer hybrid peptide vaccine to induce an immune response in patients with advanced colorectal cancer (45). In previous reports on neoantigen vaccines, published by other researchers, long peptides of 14–35 amino acids, containing neoepitopes with high affinity for HLA class I molecules, have been used for treatment (12, 16). In the first clinical trial of a neoantigen vaccine, Ott et al. reported that long synthetic peptides containing class I-restricted neoantigens induced responses not only by CD8^+^ but also by CD4^+^ T cells (12)., As expected, they found that long peptide-type vaccines containing class I-restricted neoantigen epitopes elicited more robust immune responses in CD4^+^ T cells than in CD8^+^ T cells. Although their study did not show the mechanism, the long peptide containing neoepitope seemed to activate MHC class II-restricted helper T cells.

Unlike the design of vaccines reported in these previous papers, in the design of the neoantigen vaccine reported here, the helper antigen peptide with an affinity for HLA class II HLADRB1 was initially selected, and a long peptide containing a neoantigen epitope with a strong affinity for HLA class I was then selected as a hybrid neoantigen. To the best of our knowledge, no such neoantigen design as that described here has been reported to date. Moreover, this is the first report of the analysis of cases receiving a neoantigen vaccine using a helper neoantigen peptide with an affinity for class II HLADRB, including a neoantigen epitope, with HLA class I affinity. Our findings suggest a design for future neoantigen vaccines and provide valuable data for future clinical trials of neoantigen vaccines.

The detailed mechanism by which hybrid neoantigen peptides are taken up by DCs and how peptides bound to class II and class I molecules are presented to CD4 helper T cells and CD8 cytotoxic T cells, respectively, remains unclear. Since we selected a neoantigen long peptide with strong affinity for class II molecules as a hybrid neoantigen, at least part of the hybrid peptide may directly bind to class II molecules on the surface of DCs *in vitro* (46). Long peptides that do not bind to class II molecules may also be taken up into DCs, degraded, and processed into a new peptide-MHC complex intracellularly and then translocated to the DC surface for antigen presentation (36, 47). Thus, the class I binding epitope is cross-presented to CD8^+^ T cells, and the hybrid neoantigen peptide, with a solid affinity for class II molecules, binds to class II intracellularly, directly or indirectly, thereby presenting antigens to helper T cells. Although hybrid neoantigen peptides with a strong affinity for class II molecules are assumed to bind to class II intracellularly, directly or indirectly, further analysis of the antigen presentation mechanisms of long peptides with high affinity for class II molecules is required.

## Conclusion

This study described the possible immune mechanisms underlying the efficacy of an intranodal neoantigen hybrid peptide-pulsed DC vaccine, as determined by *ex vivo* analyses of patient immune cells.

## Data availability statement

The human sequence data generated in this study are not publicly available due to patient privacy requirements but are available upon reasonable request from the first author or corresponding author. Other data generated in this study are available within the article and its supplementary data files.

## Ethics statement

Cell processing, neoantigen examination, immunotherapy procedures, and immunological analysis were approved by the ethics committee of our institution (FGCC227 EC004), with the patients’ written informed consent for the procedure based on type 3 regenerative medicine under the Japanese Law for Ensuring the Safety of Regenerative Medicine. Written informed consent was obtained from the individual(s) for the publication of any potentially identifiable images or data included in this article.

## Author contributions

SM and TSM conceptualized the study. HO supervised the study. SY, PYY, and KK predicted neoantigens and synthesized neoantigen peptides. SM, MU, NK, HT, KT, and SN performed the experiments, analyzed the data, and created the figures and tables. SM, MU, NK, HT, KT, and SN performed FACS analysis. YN designed *in silico* platforms and supervised neoepitope prediction and synthesis. SM and TSM wrote the manuscript. HO, MN, TK, TFM, MK, and YN supervised the study and discussed the data. All authors contributed to the article and approved the submitted version.

## Supplementary material

Supplementary Figure 1 Putative mechanisms of the intranodal hybrid neoantigen-pulsed DC vaccineDCs pulsed with neoantigen peptides (including class I and class II hybrid neoantigen peptides) were precisely injected into the lymph nodes under ultrasound guidance, and the DCs into which the hybrid neoantigen peptide was incorporated activated CD4^+^ T and CD8^+^ T cells via cross-presentation. Neoantigen-reactive T lymphocytes proliferated and disseminated via the efferent lymphatic ducts in the microenvironment of lymph nodes and surrounding lymph nodes.
