# Supplementary figures and images for: Immunological analysis of hybrid neoantigen peptide encompassing class I/II neoepitope-pulsed dendritic cell vaccine
Source: Front Immunol. 2023 Oct 10;14:1223331. doi: 10.3389/fimmu.2023.1223331 (PMC10595142; doi:10.3389/fimmu.2023.1223331)

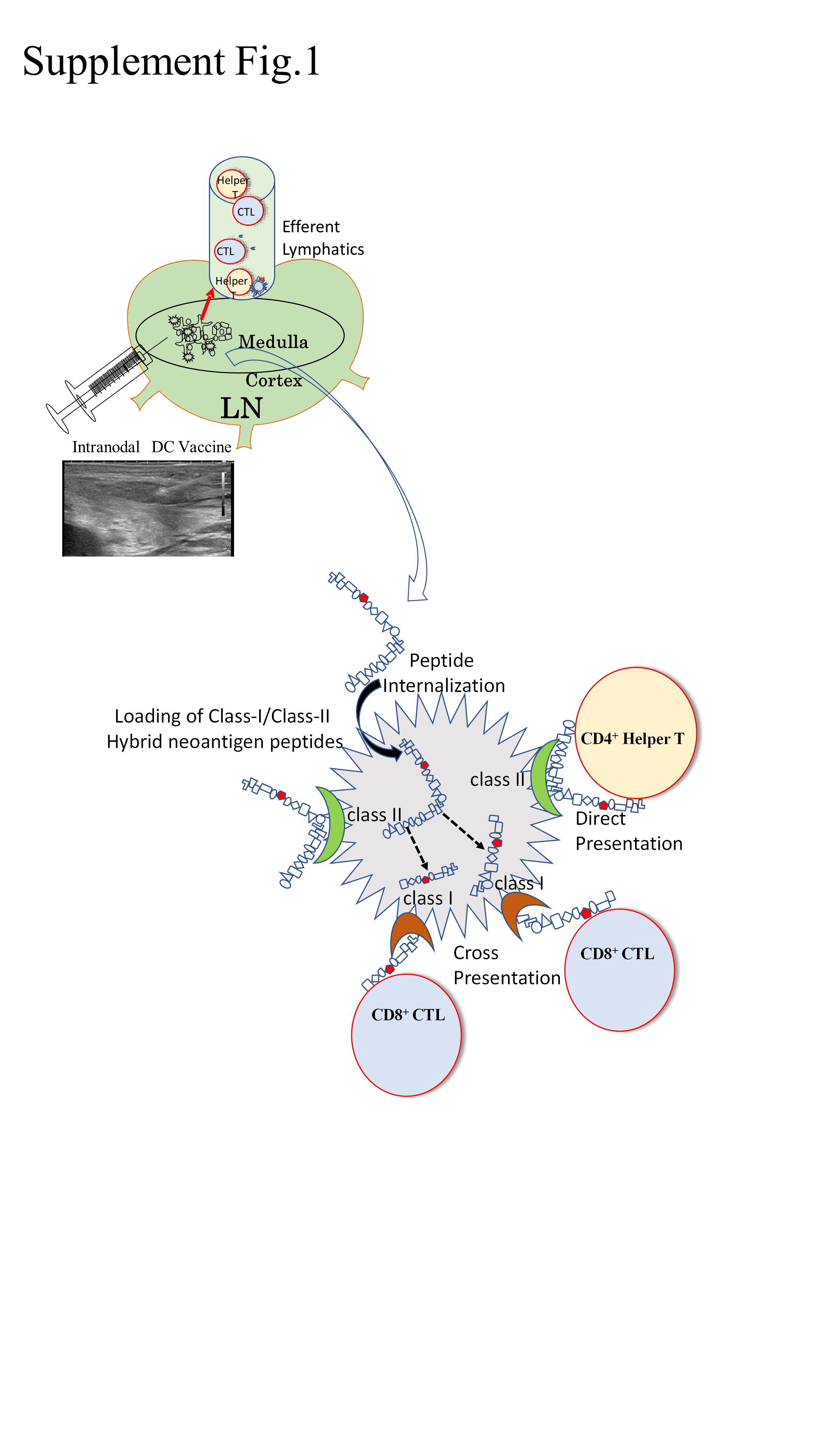

Supplement: Supplementary Figure 1 — Putative mechanisms of the intranodal hybrid neoantigen-pulsed DC vaccineDCs pulsed with neoantigen peptides (including class I and class II hybrid neoantigen peptides) were precisely injected into the lymph nodes under ultrasound guidance, and the DCs into which the hybrid neoantigen peptide was incorporated activated CD4+ T and CD8+ T cells via cross-presentation. Neoantigen-reactive T lymphocytes proliferated and disseminated via the efferent lymphatic ducts in the microenvironment of lymph nodes and surrounding lymph nodes. [file Image_1.jpeg]
